# A comparison of relative stopping power derived by fast kV‐switching dual‐energy CT, single‐energy CT, and proton CT in phantom and tissue

**DOI:** 10.1002/mp.70600

**Published:** 2026-08-05

**Authors:** Hazel Wang, Yanling Qu, Yang Li, George Dedes, Maggie Stauffer, Robert Johnson, Paul Deak, Mark Pankuch

**Affiliations:** ^1^ Medical Physics, Northwestern Medicine Warrenville Illinois USA; ^2^ CT Engineering GE Healthcare Beijing China; ^3^ Department of Medical Physics LMU Munich Munich Germany; ^4^ Santa Cruz Institute for Particle Physics University of California at Santa Cruz Santa Cruz California USA; ^5^ Research, GE Healthcare Glattbrugg Switzerland

**Keywords:** DECT, proton CT, proton therapy

## Abstract

**Background:**

The accuracy of relative stopping power (RSP) is a critical aspect for safe treatment planning in proton therapy. Dual‐energy CT (DECT) has the ability to estimate RSP more accurately than conventional single‐energy CT (SECT) due to its improved material separation. RSP accuracy is commonly determined in homogeneous phantom material as it can be more challenging to determine in heterogeneous biological tissues. Proton CT (pCT) is a modality that directly determines RSP and can be used as a comparative benchmark for evaluating the RSP accuracy of DECT and SECT derived values across homogeneous and heterogeneous material.

**Purpose:**

In this study, DECT was compared to SECT in both homogeneous phantom inserts of known RSP and heterogeneous tissue samples of unknown RSP using pCT as a quantitative reference. The purpose of this study was to assess the ability of DECT to improve RSP estimation over SECT in unknown RSP of heterogeneous tissues.

**Methods:**

A variety of homogeneous phantom inserts were scanned using pCT, DECT, and SECT. The estimated RSP from each imaging modality was compared to the RSP of each insert directly measured using a multilayer ionization chamber (MLIC). Scans of heterogeneous tissue samples were also acquired using pCT, DECT, and SECT. The images from each modality were registered and a variety of tissue regions of interest were contoured. The percent difference of DECT and SECT derived RSP values compared to pCT was calculated across both homogeneous phantom and heterogeneous tissue regions and compared.

**Results:**

The mean absolute percentage error for the inserts compared to the known RSP was 1.6% ± 1.6% in pCT, 1.1% ± 1.6% in DECT, and 4.6% ± 6.2% in SECT. For inserts with relative electron density greater than 0.93 and excluding true water and aluminum, DECT average percent difference compared to pCT was −0.5% ± 0.02% while in SECT it was −1.5% ± 0.07%. In biological tissue, excluding enamel, the average percent difference for DECT compared to pCT was −0.3% ± 1.1%, while for SECT the percent difference compared to pCT was 0.6% ± 3.0%.

**Conclusions:**

Fast kV‐switching DECT obtained smaller RSP deviations from pCT compared to SECT in homogeneous tissue‐equivalent phantom inserts. In heterogeneous tissue samples, DECT maintained improved RSP accuracy compared to SECT.

## 1 INTRODUCTION

Protons can be highly precise in delivering the therapeutic dose to the targeted area while limiting the dose to surrounding healthy tissue. This is due to the characteristic depth dose curve for protons, which deposits relatively low dose at the entrance and the maximum dose at the end of its range. The steep fall‐off at the distal end, the Bragg peak, results in a sharp dose gradient with no dose deposited beyond the targeted area. This leads to decreased radiation dose to the surrounding healthy tissues which could reduce the likelihood of treatment‐related toxicities. Because of range uncertainties in the Bragg peak's depth, distal and proximal margins must be added to the target to ensure adequate target coverage. Proton‐specific planning margins in the beam direction are typically in the range of 3.5% of the incident proton range.^1^, ^2^, ^3^, ^4^, ^5^ Depending on the incident proton energy, this translates to an additional margin of 3–11 mm into distal healthy tissue which negates some of the therapeutic precision that proton therapy aims to provide. Reducing the distal margin by reducing range uncertainty is therefore highly desirable.

There are a number of factors that contribute to the uncertainties in proton therapy planning. The largest uncertainty is the conversion of CT number from the treatment planning CT to the relative stopping power (RSP) needed for proton plan calculation.^1^, ^2^, ^3^, ^4^ The CT number is based on the linear attenuation coefficient of a material, and is a function of the material's effective atomic number ($Z_{\mathrm{eff}}$), mass density, and the photon energy.^6^ When using single‐energy CT (SECT), different materials with different relative electron density (RED) can have identical CT numbers which incorrectly convert to identical RSP values for proton planning.^1^, ^2^, ^3^, ^4^, ^7^ In addition, there is also uncertainty in the creation of the CT number to RSP conversion curve which is based on the stoichiometric calibration of tissue‐equivalent phantom. Actual tissue material may not fall on this calibration curve due to the differences in chemical composition, density, and heterogeneity.^3^, ^4^, ^5^

One modality which measures RSP directly and eliminates the uncertainty from the CT number conversion is proton CT (pCT).^8^, ^9^, ^10^, ^11^, ^12^, ^13^, ^14^ The reconstructed image of pCT is based on the energy loss of transmission protons through the object where the resulting reconstructed voxels represent a 3‐D matrix of each material RSP.^8^, ^9^ In pCT, with knowledge of the starting energy and measurement of the residual transmission energy captured at the detectors, the water equivalent path length (WEPL) can be back‐projected to reconstruct the RSP of the material being imaged.^7^, ^8^ In this way, pCT derives the RSP of a material directly. Work is still in progress in improving the design and reconstruction characteristics of a pCT system for clinical use and as a result, it is not yet a practical modality that can be clinically used for patients.^8^, ^9^, ^10^, ^11^, ^12^, ^13^, ^14^, ^15^

A modality that is becoming more clinically available is dual‐energy CT (DECT). Unlike SECT, which utilizes a single x‐ray energy spectrum, DECT reconstructs data acquired from both a low‐ and a high‐energy spectra. With CT image sets acquired at two different energy spectra, materials with different densities can be separated and identified based on the linear attenuation coefficient at those energies.^6^ This allows for the reconstruction of RED and $Z_{\mathrm{eff}}$ maps from DECT data. DECT has been shown to accurately calculate RSP based on the extraction of RED and $Z_{\mathrm{eff}}$ information with the use of homogeneous phantom material.^16^, ^17^, ^18^, ^19^, ^20^, ^21^, ^22^, ^23^ In this study, an ultrafast kV‐switching DECT was evaluated for RSP accuracy in both tissue‐equivalent and biological tissue and compared to SECT. A pCT was used to obtain RSP values of both homogeneous and heterogeneous media. The absolute accuracy of a pCT system was first compared to known RSP in homogeneous phantom and then used as a comparator for RSP evaluation in heterogeneous material.

## 2 METHODS

### 2.1 MLIC reference RSP measurements

**FIGURE 1:**
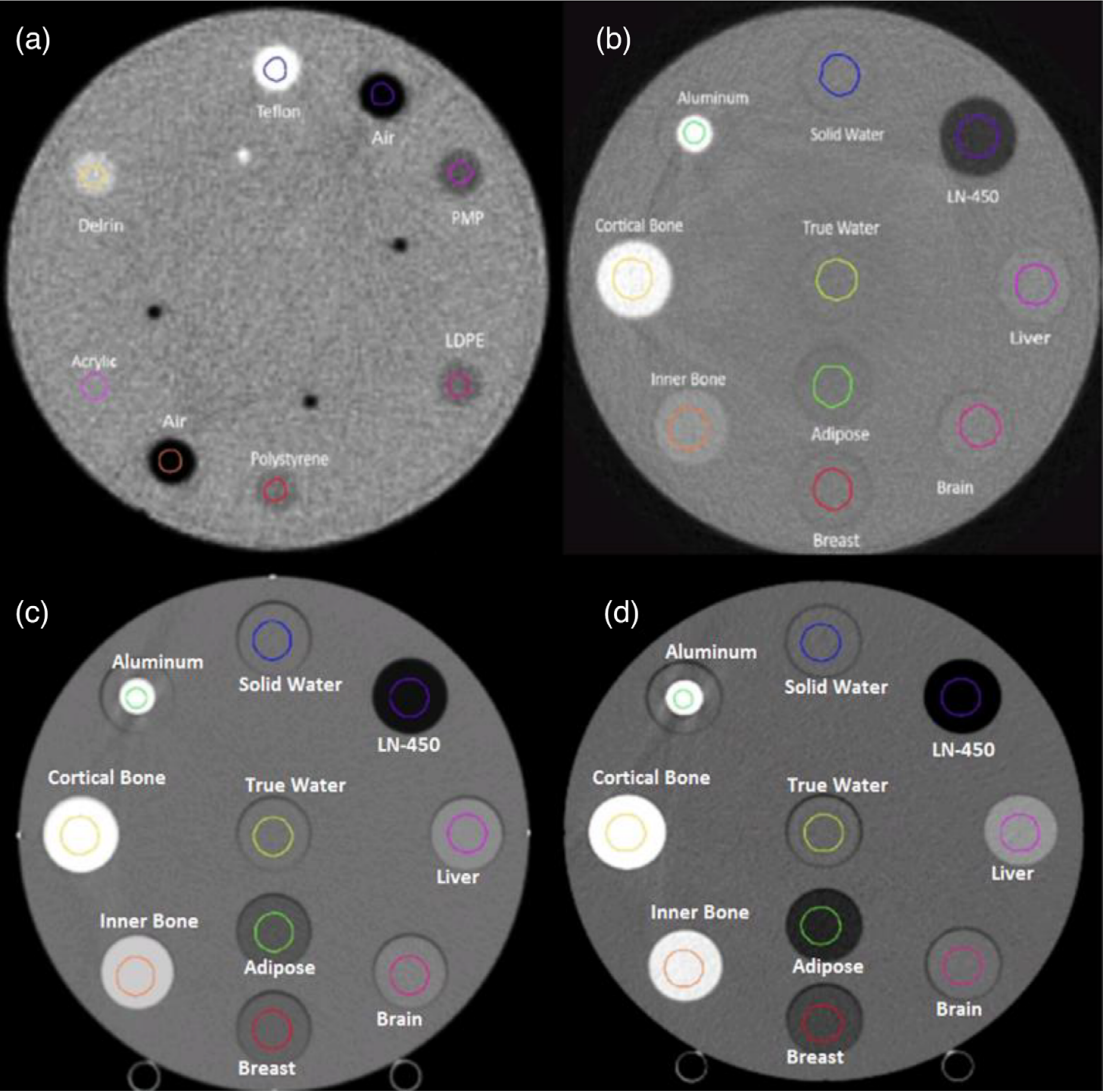
Scan images of the phantoms and ROIs (a) pCT of CTP404 phantom (b) pCT of Configuration 1 which consists of Sun Nuclear inserts (c) DECT of Configuration 1 (d) SECT of Configuration 2 containing Gammex inserts.

The CTP404 module (The Phantom Laboratory, Salem, New York), is a commonly used phantom for CT Quality Control. This phantom is a 15 cm diameter polystyrene phantom that contains 6 plastic inserts ranging from RED 0.853–1.868. The insert RSP and uncertainty was referenced from Giacometti et al.^24^ The second phantom used was composed of HE CT solid water and is a 20 cm diameter head‐sized phantom (Sun Nuclear Corporation, Middleton, WI, USA) that contains holes for inserting different inserts. In one configuration (Configuration 1 of Figure 1), 9 tissue‐equivalent phantom inserts 16.5 cm in length, which were part of the Advanced Electron Density Phantom, were arranged in the phantom to ensure adequate range for the proton imaging beam. In a second configuration (Configuration 2 of Figure 1), similar inserts 4 cm in length from the Gammex RMI 467 model were arranged to match Configuration 1. These inserts ranged from RED 0.44–1.78. Figure 1 shows the CTP404, Configuration 1 and Configuration 2 phantoms.

The determination of the reference RSP and uncertainty for each insert was previously established using water equivalent thickness (WET) measurements with a multilayer ion chamber (MLIC; “Zebra”, IBA, Louvain‐la‐Neuve, Belgium).^20^ The MLIC consists of a stack of 180 parallel plate ionization chambers that is calibrated to correspond to a known water equivalent depth to within ± 0.5 mm.^25^ In this process, the range is defined as the water equivalent depth at the 90% distal fall‐off of the percent depth dose (PDD). This defined range is the value that is used clinically. The WET of each insert was determined using a 1 cm × 1 cm proton beam of 187 MeV by measuring the range with the MLIC with and without the insert in the proton beam. The change in range is the WET. The RSP is defined as the WET divided by the physical thickness of the insert.^2^, ^19^ To ensure full traversal of the high density bone inserts, 212 MeV was used to provide a well‐defined distal fall‐off for accurate range determination. For the aluminum insert, because the aluminum core was only a diameter of 0.5 inches and because the WET of the insert was longer than the available range of the proton beam, the RSP could not be determined with the MLIC. Instead, a theoretical reference was used for the aluminum insert. The aluminum reference RSP was calculated with the Bethe‐Bloch equation described in Section 2.4 Equation 1 using the vendor specified RED of 2.36, atomic number 13, proton energy of 175 MeV, and I‐value of 166 eV.^26^

### 2.2 Proton CT scans

The pCT images used in this study were acquired with a second generation prototype scanner designed and built by the Loma Linda University and University of California at Santa Cruz described as a phase II scanner.^27^ This system consists of front and rear tracking detectors as well as a scintillator detector to measure the residual energy of the protons.^9^ The accuracy of pCT was verified by comparing the calculated RSP of several inserts with MLIC measured RSP described in 2.1. In prior works, the CTP404 was imaged using the same pCT scanner utilized in this study.^11^ Each phantom was placed on a platform that rotated in a uniform 200 MeV proton broad beam for imaging. For the phase II pCT, the system was calibrated and images post‐processed, mapping the energy of each proton into WEPL.^28^ The resulting reconstructed pCT imaging dose was 1–2 mGy and contained a 1 mm × 1 mm × 1 mm grid of 250 × 250 voxels which directly represented a 3‐D mapping of the RSP of the phantom.^12^

### 2.3 SECT and DECT scans

Configuration 1, Configuration 2, and CTP404 phantoms were helically scanned with SECT (GE Revolution, GE Medical Systems, Milwaukee, WI, USA) and reconstructed without metal artifact corrections. SECT scanning protocol utilized 120 kVp, 165 mA, Large Body scanning field of view (SFOV), display field of view (DFOV) 35 cm, 0.992 pitch, 80 mm detector width, 0.5 s rotation time, 2.5 mm thickness. Although a head‐sized phantom was used, all scanning protocols at our center utilize the largest SFOV to ensure all devices that are used for treatment and appear in the beam path are included in the scan. The CTDI_vol,32cmbody_ reported on the scanner was 34.77 mGy. The fast kV‐switching DECT consists of a single source and a single detector that rapidly alternates between 80 kVp to 140 kVp at sub‐millisecond speed for a cycle time of 0.25 ms.^29^, ^30^ The phantoms were rescanned with DECT utilizing an available default Gemstone Spectrum Imaging (GSI) protocol that closely matched SECT scanning parameters and dose. This protocol utilized 485 mA, Large Body SFOV, DFOV 35 cm, 0.992 pitch, 40 mm detector width, 0.8 s rotation time, 2.5 mm thickness. The CTDI_vol,32cmbody_ reported by the scanner was 36.98 mGy.

### 2.4 Biological tissue image acquisition

**FIGURE 2:**
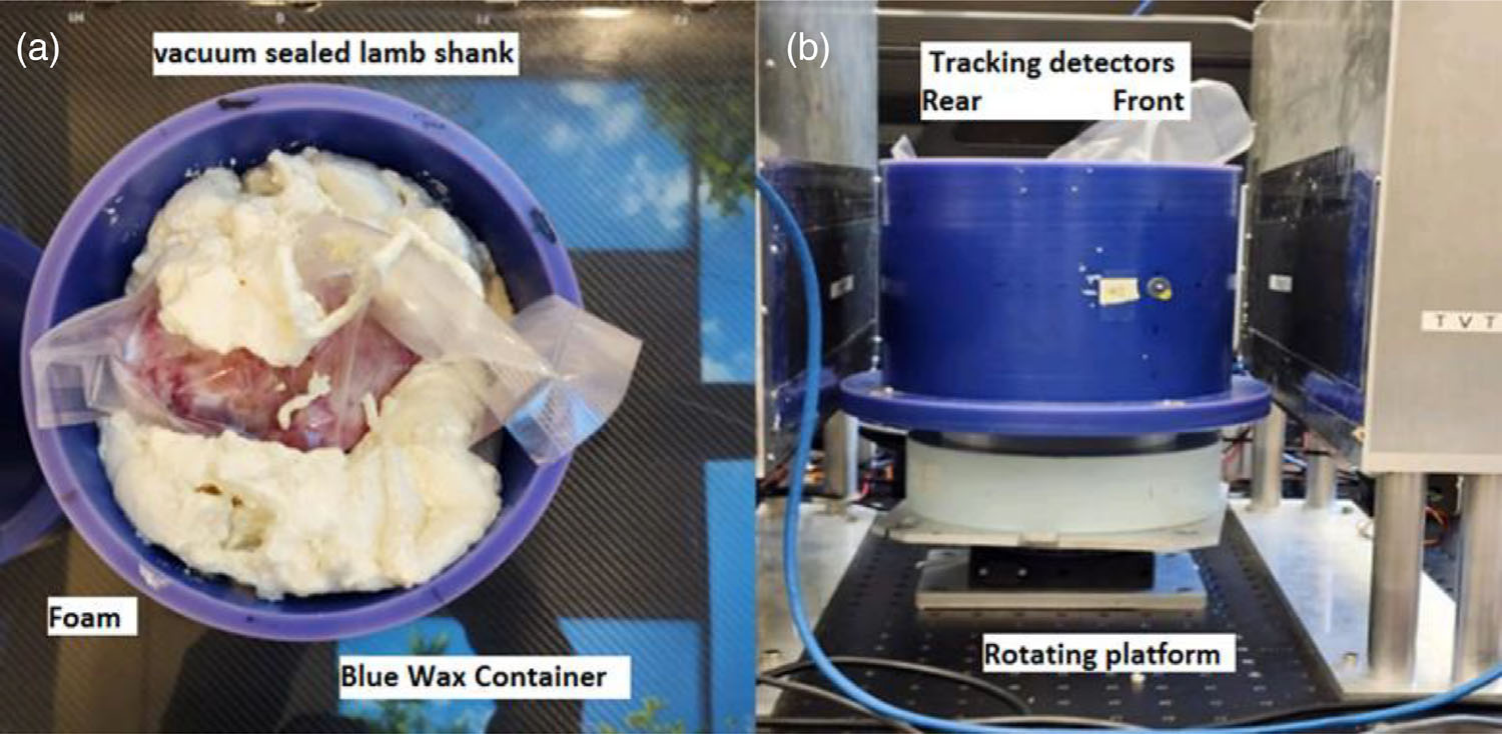
(a) Top view of the immobilized lamb shank in blue wax container (b) pCT setup for biological tissue scan.

A fresh post‐mortem lamb head and lamb shank were individually vacuum sealed and immobilized with foam into 21 and 18 cm diameter blue wax containers, respectively. The blue wax container used was 1.0 cm thick with known RSP of 0.977. The blue wax RSP was validated previously with measurements using MLIC and is the value used clinically in the treatment planning system. A pCT was acquired for each sample with the same settings as the phantom scans described in Section 2.2. In order to ensure similar quality and integrity of the tissue samples, scans of DECT and SECT were performed immediately after scans with pCT using the CT scanning parameters described in Section 2.3. The time between scanning was no longer than 5 min. Each container was scanned in the upright position to mimic the exact orientation of pCT scan. Figure 2 depicts the immobilization for the animal samples inside the containers and the upright orientation scanned on pCT. To confirm that no additional CT artifacts were introduced due to orientation, each container was tipped 90 degrees and rescanned.

### 2.5 Data analysis

GE provided reconstructed RED, $Z_{\mathrm{eff},}$ and RSP maps for each of DECT scans. RED and $Z_{\mathrm{eff}}$ were reconstructed using a proprietary algorithm based on Näsmark & Anderson.^31^ Proton RSP was calculated according to the Bethe‐Bloch equation

(1) $$RSP=\frac{\rho_{e,m}\left[ln\left(\frac{2m_{e}c^{2}\beta^{2}}{\left(1-\beta^{2}\right)}\right)-\beta^{2}-lnI_{m}\right]}{\rho_{e,w}\left[ln\left(\frac{2m_{e}c^{2}\beta^{2}}{\left(1-\beta^{2}\right)}\right)-\beta^{2}-lnI_{w}\right]}\;$$

where $m_{e}$ is the rest mass of the electron, c is the speed of light in vacuum, $\rho_{e,m}/\rho_{e,w}$ is RED, $I_{m}$ is the mean excitation energy of the material, m, $I_{w}$ for water is 75.3 eV, and β is speed of 200 MeV protons relative to c. The *I*‐value of the material can be determined by the Bragg‐Additivity Rule

(2) $$ln\;I_{m}=\frac{\sum_{j=1}^{J}\frac{w_{j}Z_{j}}{A_{j}}lnI_{j}}{\sum_{j=1}^{J}\frac{w_{j}Z_{j}}{A_{j}}}\;$$

where $w_{j}$ is the mass weight fraction, $Z_{j}$ is the effective atomic number, and $A_{j}$ is the mass number.

**FIGURE 3:**
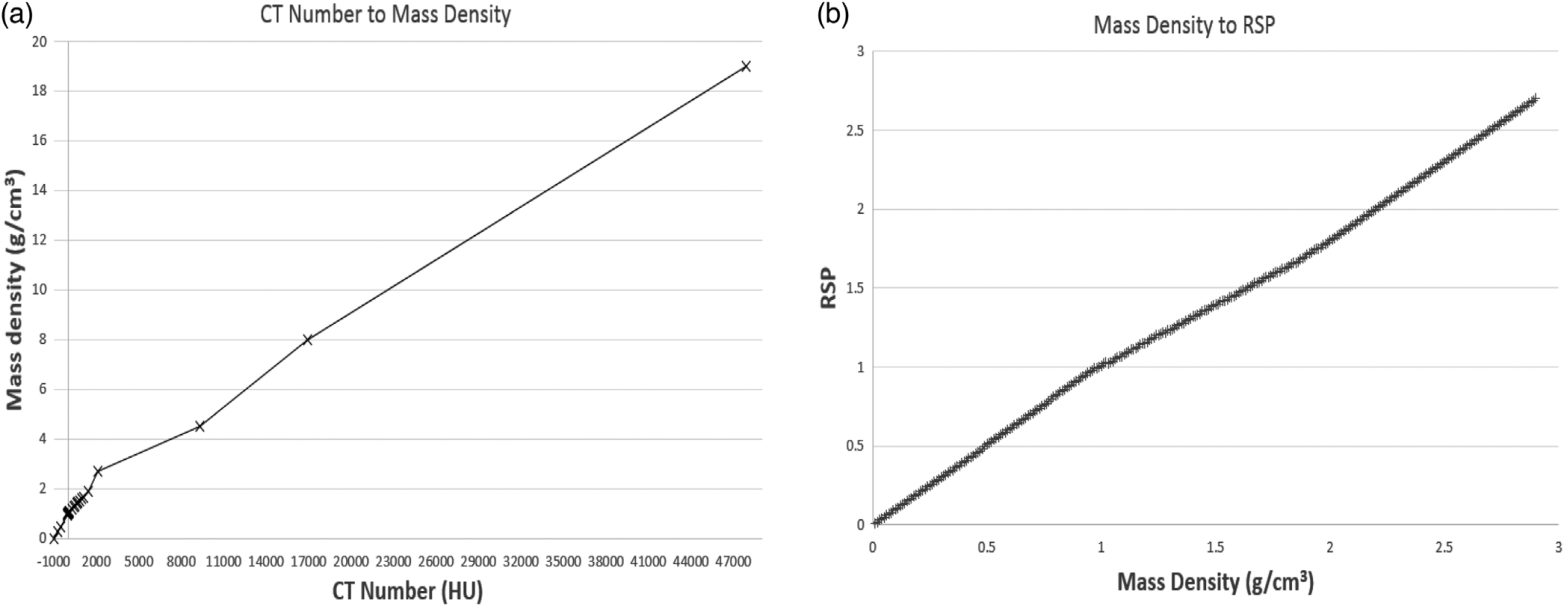
CT number to RSP conversion. “x” denotes calibration points interpolated with a line. (a) CT number to mass density conversion curve (b) Mass density to RSP conversion curve.

Images from pCT were rigidly registered to their corresponding DECT and SECT images. For the Configuration 1 and Configuration 2 phantoms, a cylindrical ROI including at least 60% of the volume was centered for each insert in order to avoid the edges. The ROI diameter was 1.5 cm and length was 2.7 cm, giving a volume of 4.7 cm^3^. The ROI diameter for the aluminum insert was smaller (0.75 cm/ 0.3 inches) due to the metal being a 0.5 inch core. The length of the cylinder was 5.3 cm for a total volume of 2.5 cm^3^. For the CTP404, the volume for each cylindrical ROI was 0.2 cm^3^. For each insert, the ROI contoured on pCT was rigidly copied onto DECT and SECT. Figure 1 displays the resulting scans for pCT, DECT, and SECT and the ROIs drawn on each phantom insert. The mean RSP of pCT and DECT was recorded. For SECT evaluation, the CT number of each voxel within the ROI was converted to RSP using stoichiometric calibration and the mean RSP value was recorded.^4^ The stoichiometric calibration curve used for SECT HU‐RSP conversion is shown in Figure 3. Tissue‐equivalent phantom inserts from Configuration 1 and 2 were classified based on their RED value as either lung (0.28–0.44), fat (0.93–0.97), muscle (1.02–1.08), water (0.99–1.00), or bone (1.09–1.78).

**FIGURE 4:**
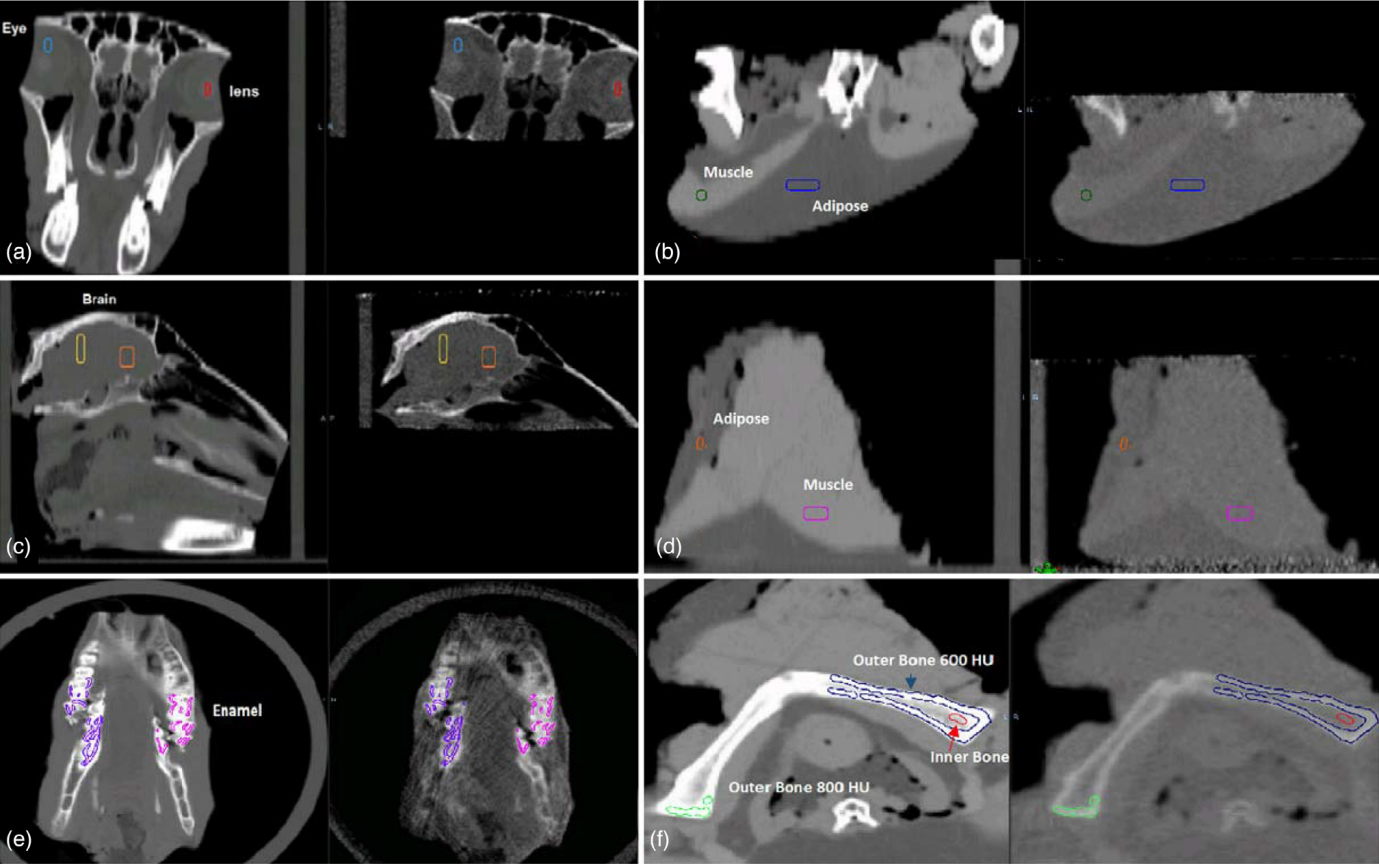
(a–f) shows the resulting DECT (left) and pCT scans (right) of the lamb head (A,C,E) and lamb shank (B,D,F) with sample ROIs. The ROIs were initially contoured on SECT and verified on DECT and pCT. A. Blue = Eye, Red = Lens B. Green = Muscle, Blue = Adipose C. Yellow = Posterior Brain, Orange = Anterior Brain D. Orange = Adipose, Magenta = Muscle E. Purple = Enamel Right, Magenta = Enamel Left F. Green = Outer Bone 800 HU, Blue = Outer Bone 600 HU, Red = Inner Bone.

For animal samples, a variety of tissue regions were contoured on the lamb head and lamb shank images. Figure 4a–f shows the various ROIs sampled in DECT and pCT. To ensure accuracy in tissue delineation between the modalities, ROIs were selected away from high gradient areas of different tissues. All ROIs were delineated using SECT. They were then visually verified in the registration with DECT and pCT to ensure there was no overlap with other tissues and remained away from edges. For ROIs in highly heterogeneous areas of hard and soft bone, CT number thresholds were utilized. This segmentation allowed only the areas within a specified boundary that is above a specified CT number to be included in the ROI. A threshold of 600 HU was selected for one sample of cortical bone. A second ROI for cortical bone was set to a threshold of 800 HU. In enamel, the threshold was set to 2000 HU. Figure 4e–f depicts the segmentation as a result of thresholding on SECT dataset registered on the corresponding DECT and pCT images.

RSP accuracy compared to MLIC was quantified using percent error

(3) $$\%ERR=\frac{ROI_{\mathrm{mean}}-RSP_{\mathrm{ref}}}{RSP_{\mathrm{ref}}}\;*100\%$$

where $RSP_{\mathrm{ref}}$ is the reference RSP described in section 2.1 and $ROI_{\mathrm{mean}}$ is the RSP resulting from the mean value of the voxels in the ROI sampled. The mean percent error as well as mean absolute percent error (MAPE) were also calculated. MAPE is defined as

(4) $$MAPE=\frac{\sum \left|\%\mathrm{ERR}\right|}{n}$$

where *n* is the number of inserts evaluated.

Because the actual RSP of heterogeneous tissues cannot be determined using MLIC measurements like in homogeneous phantom, pCT was used as a comparator in the lamb tissue. While DECT and SECT estimate RSP indirectly from x‐ray attenuation, pCT directly determines RSP through proton energy‐loss measurements making it a suitable independent estimate for comparison. The RSP percent difference for DECT and SECT from pCT was calculated as

(5) $$\%\mathrm{Diff}=\frac{\;\mathrm{RS}P_{\mathrm{xCT}}-\;\mathrm{RS}P_{\mathrm{pCT}}}{1/2\left(\mathrm{RS}P_{\mathrm{xCT}}+\mathrm{RS}P_{\mathrm{pCT}}\right)}*100\%$$

where $\mathrm{RS}P_{\mathrm{xCT}}$ is the RSP value from either SECT or DECT.

## 3 RESULTS

### 3.1 RSP accuracy in phantoms

The reference values for RED, $Z_{\mathrm{eff}}$, RSP, as well as the RSP estimated based on each imaging modality, and the absolute difference and percent error of the RSP with respect to the MLIC measurements are listed in Table 1. The largest RSP percent errors in pCT were found in the low density inserts (PMP 3.5%, LN 450 5.8%, 6.0%). The percent error in pCT for all other inserts ranged from −2.8% to 2.1%. The MAPE for pCT was 1.6% ± 1.6%. With the low density and central inserts removed, the MAPE for pCT was 1.0% ± 0.8%. The largest error in DECT was aluminum at 6.0%. The percent error in the remaining inserts ranged from −2.0% to 2.7% and MAPE was 1.1% ± 1.6%. For SECT, the largest percent errors were observed in Teflon at −21%, Delrin at −15.4%, aluminum at 18%, and acrylic at −6.2%. The percent error of all other inserts in SECT ranged from −4.4% to 5% and MAPE was 4.6% ± 6.2%.

**TABLE 1:** MLIC, pCT, DECT, and SECT RSP in phantom. Inserts listed in order of increasing RED Relative Electron Density; $Z_{\mathrm{eff},}$ Effective atomic number; RSP relative stopping power; MLIC multilayer ionization chamber; pCT proton CT, DECT Dual‐energy CT, SECT Single‐energy CT; Δ Dif describes the RSP absolute difference from RSP_MLIC_; %Eff as defined in Equation 3; MAPE mean absolute percent error as defined in Equation 4; 1 SN = Configuration 1 Sun Nuclear; 2 GX = Configuration 2 Gammex.

			MLIC	pCT			DECT			SECT		
Insert	RED	$Z_{\mathrm{eff}}$	RSP	RSP	Δ Dif	%Err	RSP	Δ Dif	%Err	RSP	Δ Dif	%Err
LN 450 (1 SN)	0.44	7.578	0.477 ± 0.001	0.505 ± 0.037	0.028	5.8	0.474 ± 0.004	−0.003	−0.7	0.489 ± 0.105	0.012	2.5
LN 450 (2 GX)	0.44	7.578	0.459 ± 0.006	0.486 ± 0.040	0.027	6.0	0.451 ± 0.005	−0.008	−1.8	0.471 ± 0.112	0.012	2.7
PMP (CTP404)	0.85	5.44	0.883 ± 0.002	0.911 ± 0.040	0.028	3.5	0.881 ± 0.002	0.000	0.1	0.860 ± 0.063	−0.020	−2.3
Adipose (2 GX)	0.93	6.144	0.939 ± 0.003	0.945 ± 0.030	0.006	0.7	0.938 ± 0.005	0.000	0.0	0.949 ± 0.098	0.011	1.1
Adipose (1 SN)	0.94	6.611	0.960 ± 0.002	0.964 ± 0.030	0.004	0.4	0.954 ± 0.005	−0.005	−0.6	0.974 ± 0.095	0.014	1.5
LDPE (CTP404)	0.95	5.44	0.980 ± 0.002	0.995 ± 0.040	0.015	1.5	0.976 ± 0.002	−0.004	−0.4	0.951 ± 0.052	−0.029	−3.0
Breast (2 GX)	0.96	6.821	0.976 ± 0.003	0.987 ± 0.040	0.011	1.1	0.968 ± 0.004	−0.008	−0.8	0.987 ± 0.084	0.011	1.1
Breast (1 SN)	0.97	6.922	0.981 ± 0.002	0.995 ± 0.036	0.014	1.4	0.976 ± 0.004	−0.005	−0.6	0.989 ± 0.086	0.008	0.8
Solid Water (2 GX)	0.99	7.591	1.005 ± 0.002	1.010 ± 0.040	0.005	0.5	0.998 ± 0.004	−0.007	−0.7	1.014 ± 0.083	0.009	0.9
Solid Water (1 SN)	1.00	7.445	1.002 ± 0.002	1.011 ± 0.036	0.009	0.9	1.000 ± 0.004	−0.001	−0.1	1.010 ± 0.080	0.008	0.8
Poly‐styrene (CTP404)	1.00	5.7	1.024 ± 0.001	1.042 ± 0.040	0.022	2.1	1.028 ± 0.002	0.008	0.8	0.990 ± 0.068	−0.030	−2.9
True Water 1 (1 SN)	1.00	7.42	1.000 ± 0.000	0.979 ± 0.034	−0.021	−2.1	1.002 ± 0.006	0.002	0.2	1.001 ± 0.093	0.001	0.1
True Water 2 (1 SN)	1.00	7.42	1.000 ± 0.000	0.975 ± 0.030	−0.026	−2.6	1.002 ± 0.006	0.002	0.2	1.012 ± 0.093	0.012	1.2
Brain (1 SN)	1.02	7.633	1.028 ± 0.002	1.038 ± 0.039	0.011	1.1	1.028 ± 0.004	0.001	0.1	1.032 ± 0.084	0.004	0.4
Brain (2 GX)	1.04	6.14	1.070 ± 0.003	1.071 ± 0.040	0.002	0.1	1.048 ± 0.005	−0.021	−2.0	1.022 ± 0.081	−0.047	−4.4
Liver (1 SN)	1.05	7.613	1.059 ± 0.002	1.067 ± 0.038	0.008	0.8	1.058 ± 0.004	0.000	0.0	1.052 ± 0.078	−0.006	−0.6
Liver (2 GX)	1.06	7.592	1.080 ± 0.003	1.081 ± 0.040	0.001	0.1	1.072 ± 0.004	−0.008	−0.7	1.064 ± 0.074	−0.016	−1.5
Inner Bone (2 GX)	1.09	10.11	1.090 ± 0.003	1.091 ± 0.040	0.001	0.1	1.082 ± 0.004	−0.008	−0.8	1.124 ± 0.087	0.034	3.1
Acrylic (CTP404)	1.15	6.47	1.160 ± 0.001	1.173 ± 0.040	0.013	1.1	1.160 ± 0.002	0.000	0.0	1.088 ± 0.062	−0.072	−6.2
Inner Bone (1 SN)	1.16	10.39	1.143 ± 0.002	1.146 ± 0.038	0.003	0.2	1.142 ± 0.003	−0.001	−0.1	1.160 ± 0.093	0.017	1.5
Delrin (CTP404)	1.36	6.95	1.359 ± 0.003	1.366 ± 0.050	0.006	0.5	1.363 ± 0.002	0.003	0.2	1.150 ± 0.082	−0.210	−15.4
Cortical Bone (2 GX)	1.69	13.53	1.616 ± 0.005	1.596 ± 0.040	−0.020	−1.2	1.633 ± 0.004	0.017	1.1	1.660 ± 0.112	0.044	2.7
Cortical Bone (1 SN)	1.78	13.63	1.699 ± 0.002	1.669 ± 0.043	−0.030	−1.7	1.717 ± 0.004	0.018	1.1	1.784 ± 0.120	0.085	5.0
Teflon (CTP 404)	1.87	8.43	1.790 ± 0.002	1.781 ± 0.050	−0.009	−0.5	1.839 ± 0.003	0.049	2.7	1.423 ± 0.077	−0.367	−21
Aluminum (1 SN)	2.36	13	2.14^*^	2.082 ± 0.046	−0.183	−2.7	2.266 ± 0.009	0.001	5.9	2.538 ± 0.141	0.273	18
Aluminum (2 GX)	2.36	13	2.14^*^	2.081 ± 0.050	−0.184	−2.8	2.268 ± 0.009	0.002	6.0	2.536 ± 0.186	0.271	18
MAPE MAPE^**^				1.6% ± 1.6% 1.0% ± 0.8%			1.1% ± 1.6%			4.6% ± 6.2%		

* Calculated using Bethe‐Bloch Equation 1.

** MAPE of pCT without lung, PMP, and true water.

### 3.2 Comparison of DECT and SECT to pCT in phantoms

**FIGURE 5:**
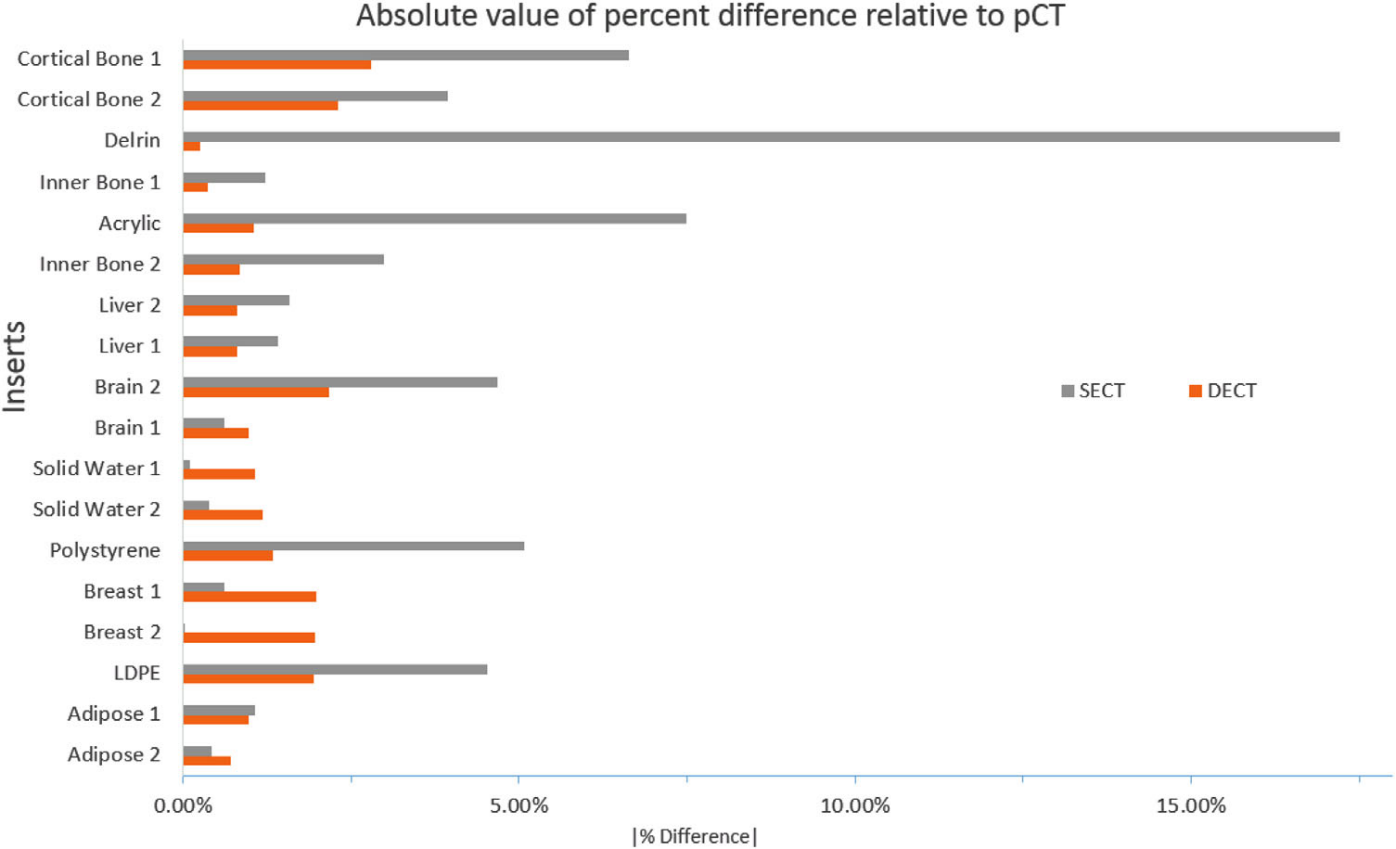
Absolute value of percent difference for the phantom inserts for SECT and DECT compared to pCT. Low density inserts, true water, and aluminum excluded due to large errors in estimation. 1–Configuration 1 Sun Nuclear Inserts, 2–Configuration 2 Gammex inserts.

Figure 5 shows the absolute value of the RSP percent difference of DECT and SECT to pCT for each phantom inserts excluding the PMP, true water, and LN 450 inserts which did not give accurate RSP estimates in pCT. In addition, due to the large error in SECT and DECT, aluminum was also removed from the figure in order to maintain a readable scale and to allow more meaningful visual comparison among the other materials. MAPE across all inserts for DECT was 2.5% ± 2.4% and SECT was 5.4% ± 6.6%. Comparing the results of each insert, DECT deviated less from pCT than SECT in most inserts.

### 3.3 Comparison of DECT and SECT to pCT in biological tissue

There were no artifacts visually noted between the images of the upright and tipped position of the containers. In DECT and SECT, the average standard deviation of the mean CT number in ROI samples of fat, eye, brain, muscle, lens, and bone between the upright and supine scans was within ± 0.003. This indicated no impact on image quality due to the container orientation.

Table 2 displays the list of ROIs and volume size drawn on the lamb head and lamb shank datasets, corresponding RED, $Z_{\mathrm{eff},}$ and RSP measurements from DECT and percent difference of RSP from DECT and SECT, to that of pCT. When comparing to the RSP of pCT, the largest RSP percent difference was seen in both sampled regions of high density enamel resulting in percent differences of 17.4% and 24.1% in DECT and 43.5% and 49.2% in SECT. Without enamel, the average RSP percent difference for all tissues was −0.3% ± 1.1% for DECT and 0.6% ± 3.0% for SECT.

**TABLE 2:** Sampled regions of lamb head and shank. RED and $Z_{\mathrm{eff}}$ as determined by DECT; %Diff relative to pCT calculated using Equation 5.

Tissue	Part	Vol (cc)	RED	$Z_{\mathrm{eff}}$	pCT_RSP_	DECT_RSP_	DECT %Diff	SECT_RSP_	SECT %Diff
Fat	Shank	0.5	0.941	6.110	0.97	0.96	−1.5%	0.96	−1.6%
Fat	Shank	0.1	0.943	6.063	0.97	0.96	−1.3%	0.95	−2.3%
Fat	Head	0.1	0.951	6.229	0.97	0.97	−0.4%	0.97	0.1%
Eye R	Head	0.2	1.016	7.427	1.02	1.02	−0.6%	1.02	0.1%
Eye L	Head	0.2	1.016	7.570	1.02	1.02	−0.4%	1.02	0.2%
Brain	Head	3.8	1.039	7.474	1.04	1.04	0.0%	1.03	−0.7%
Brain	Head	1.3	1.040	7.585	1.05	1.04	−0.7%	1.03	−1.3%
Muscle	Head	0.3	1.060	7.657	1.06	1.06	0.0%	1.05	−1.2%
Muscle	Shank	0.2	1.062	7.429	1.06	1.07	0.7%	1.05	−0.7%
Muscle	Head	0.2	1.065	7.597	1.05	1.06	1.0%	1.05	−0.2%
Muscle	Shank	1.4	1.066	7.406	1.07	1.06	−0.4%	1.05	−2.0%
Lens	Head	0.1	1.128	7.679	1.15	1.13	−1.7%	1.09	−4.9%
Inner Bone	Shank	0.04	1.136	10.50	1.13	1.12	−1.4%	1.16	1.9%
Inner Bone	Shank	0.2	1.173	10.86	1.18	1.15	−2.5%	1.19	0.7%
Cortical Bone	Shank	7.9	1.511	12.10	1.41	1.43	1.4%	1.50	6.0%
Cortical Bone	Shank	4.6	1.562	12.31	1.48	1.47	−0.2%	1.51	2.4%
Cortical Bone	Shank	4.5	1.606	12.46	1.49	1.51	1.6%	1.57	5.6%
Cortical Bon	Shank	3.1	1.641	12.55	1.54	1.54	−0.1%	1.59	2.9%
Cortical Bone	Head	0.1	1.663	12.92	1.59	1.58	−0.4%	1.59	0.2%
Cortical Bone	Shank	0.04	1.860	13.40	1.73	1.76	1.4%	1.87	7.7%
Enamel	Head	3.1	1.988	13.55	1.64	1.95	17.4%	2.55	43.5%
Enamel	Head	4.0	2.006	13.61	1.54	1.97	24.1%	2.55	49.2%

Table 3 summarizes the MAPE in tissue‐equivalent phantom and in biological tissue between DECT and SECT compared to pCT based on the tissue categories lung, fat, muscle, water and bone as defined in section 2.5. In nearly all categories, DECT outperformed SECT. The largest RSP errors for both DECT and SECT was in bone for phantom and tissue.

**TABLE 3:** RSP MAPE: Phantom comparison to MLIC and tissue comparison to pCT.

	pCT	DECT		SECT	
	phantom	phantom	tissue	phantom	tissue
plastic	1.5% ± 1.1%	0.7% ± 1.0%	—	8.4% ± 7.7%	—
lung	5.9% ± 0.2%	1.8% ± 0.8%	—	2.6% ± 0.1%	—
fat	0.9% ± 0.5%	0.5% ± 0.3%	1.0% ± 0.6%	1.1% ± 0.3%	1.3% ± 1.1%
water	0.7% ± 0.3%^*^	0.4% ± 0.4%^*^	0.5% ± 0.1%	0.8% ± 0.0%^*^	0.1% ± 0.1%
muscle	0.5% ± 0.5%	0.7% ± 0.9%	0.5% ± 0.4%	1.7% ± 1.9%	1.0% ± 0.6%
bone	0.8% ± 0.8%	0.8% ± 0.4%	1.2% ± 0.8%^**^	3.1% ± 1.5%	3.6% ± 2.6%^**^

* Evaluated without true water insert.

** Evaluated without enamel.

## 4 DISCUSSION

Because pCT technology is still in development, pCT image quality is not as advanced as x‐ray CT and its limitations have been described in detail.^12, 15, 32^ Low‐density material can present streak artifacts due to the small energy loss of protons when traversing it. The fewer interactions in less dense material may result in WEPL/RSP values further from the most probable WEPL/RSP expected by the stochastic description of energy loss via the Bethe‐Bloch equation used during image recontruction.^14^ Ring artifacts are also known to stem from this particular energy detector prototype design due to the cylindrical shape of the phantom. The ring artifacts were partially mitigated using specific correction algorigthms.^12^ Another artifact specific to this second generation pCT is large RSP underestimation at the center of the image.^12^ This is evident in the measurement of RSP at the center true water insert of Configurations 1 and 2, which had a percent difference of −2.1% and −2.6%, respectively. Previous studies report pCT accuracy to be within 1%–1.5%.^8^, ^9^, ^10^, ^11^, ^12^ Because the results of the true water value in pCT was outside the expected range and its inclusion in the analysis distorts the average, the MAPE results were presented with and without the true water insert, due to its center location in the phantom. Its exclusion was applied consistently across DECT and SECT in Table 3. Dedes et al. reported a MAPE of < 1% using the CTP404.^12^ In this study, all phantom inserts resulted in a MAPE of 1.6% ± 1.6%. Excluding low density inserts and the central water insert resulted in a MAPE of 1.0% ± 0.8%. Therefore, the calibration of pCT was found to be appropriate except in low density regions with RED < 0.93.

The voxel resolution of pCT is 1 mm while DECT and SECT was 0.68 mm. Because of the higher resolution and contrast of SECT images, contours were drawn initially on SECT and modified on pCT to ensure the regions were within the intended boundaries of the tissue. However, the difference in resolution resulted in more volume averaging seen with pCT especially in smaller heterogeneous structures such as enamel and outer bone. Thresholding was used in these regions to ensure regions of interest that were drawn remained above specified CT number boundaries. To evaluate the ROI in cortical bone, the difference of min and max RSP was 0.3 for both DECT and pCT. However, in separate regions of enamel the difference of min and max RSP was 0.7 in DECT while in pCT it was 1.3. Therefore, RSP evaluation in enamel was not included in the overall average percent difference due to inaccuracy caused by large volume averaging. Improvement in imaging low density materials and increase in resolution of pCT could further improve the RSP measurements in smaller volume ROI evaluations.

In DECT, the average RSP percent error in tissue‐equivalent material (excluding aluminum and plastic inserts from CTP404) was ‐0.42% ± 0.82%. This is consistent with the consolidated findings by Bär et al. who used the Gammex 467 phantom and found that the RSP mean percent error was within ± 0.57% for most RSP estimation methods.^16^ When applying the methods to human reference tissues the mean percent error was found to be within 0.3%. However, the human reference tissues were a simulation of the average composition of each tissue for humans and may still have a large variance for the actual sample being examined and scanning conditions. In another study by Yang et al., a comparison of SECT to DECT RSP calculations in human reference tissues resulted in RMS error of 0.89% in SECT and 0.26% in DECT.^22^ In this study, actual post mortem biological tissue was examined and the mean percent difference excluding enamel was 0.6% in SECT and −0.3% in DECT.

In another study by Bär et al., the authors compared DECT, SECT, pCT and HeCT in bovine and porcine tissue samples.^33^ They found that DECT performed slightly better than pCT with a RMSE of 0.75% ± 2.8% in DECT and 1.19% ± 2.81% in pCT. Both of these modalities outperformed SECT which had a RMSE of 3.10% ± 2.88%. Although tissue samples were inhomogenous, the geometry was not complex. The samples were uniform regions fit into fixed cylindrical tubes. The RSP was determined using peak detection measurements in water. Their results are consistent with the results of pCT, DECT, and SECT in homogeneous tissue‐equivalent material of known RSP found in Table 1.

In a study by Wohlfahrt et al., RSP prediction of SECT and DECT was compared in an anthropomorphic head phantom.^21^ Although this was not actual biological tissue, this study allowed for an evaluation in an inhomogenous complex geometry. The findings showed that the direct RSP calculated by DECT predicted RSP better than the HU to RSP conversion from SECT.

## 5 CONCLUSIONS

Due to the inaccuracy of imaging low density materials and known reconstruction artifacts of the current pCT prototype, the RSP evaluation in animal tissue was limited to sampling in uniform tissue regions with RED greater than 0.93. However, pCT served as an acceptable reference standard for comparison of RSP in unknown biological material with DECT to SECT. DECT RSP calculated in phantom and in animal tissue showed smaller deviations from pCT RSP derived values than SECT in most materials which demonstrates improved material characterization using DECT.

## CONFLICT OF INTEREST STATEMENT

The authors declare no conflicts of interest.
